# Correlation between atypical squamous cells–cannot exclude high-grade squamous intraepithelial lesion and cervical lesions: a retrospective analysis of opportunistic and organized cervical screening populations

**DOI:** 10.3389/fonc.2026.1926183

**Published:** 2026-08-14

**Authors:** Linhong Liao, Hui Cheng, Hui Su, Xi Wang

**Affiliations:** 1 Department of Pathology, Ganzhou Maternal and Child Health Care Hospital, Ganzhou, Jiangxi, China; 2 Department of Emergency, People’s Hospital of Ganzhou City, Ganzhou, Jiangxi, China

**Keywords:** artificial intelligence, cervical cancer screening, opportunistic screening, organized screening, squamous intraepithelial lesions

## Abstract

**Background:**

We compared Liquid-Based Cytologic Test (LCT) results diagnosed as atypical squamous cells–cannot exclude high-grade squamous intraepithelial lesion (ASC-H) with high-risk human papillomavirus (hrHPV) infection status and histopathological findings across two cervical screening groups. Additionally, we analyzed the clinical correlation of ASC-H with cervical lesions.

**Methods:**

This retrospective analysis included 171,688 cases. LCT specimens were divided into two groups: 31,100 from patients who underwent organized cervical screening with artificial intelligence(AI)-assisted interpretation; and 140,588 from patients who underwent opportunistic screening interpreted manually. Of the 663 cases diagnosed with ASC-H, 387 underwent both HPV testing and subsequent histopathological examination. HPV detection methods included 23-type HPV genotyping, and the Cobas 4800 assay which identifies HPV16/18 plus 12 other hrHPV subtypes.

**Results:**

The overall prevalence of ASC-H was 0.39%. Among 387 paired cases of ASC-H, the total HPV infection rate reached 84.00%, with hrHPV accounting for 97.82% of cases. Women aged <50 years exhibited more single HPV genotype infections and a significantly higher positivity rate for hrHPV than those aged ≥50 years (*p*<0.05). In 297 ASC-H samples tested by HPV23 genotyping, the most prevalent hrHPV genotypes were HPV16 (28.62%), HPV52 (21.89%), and HPV58 (17.17%). Among 90 ASC-H samples detected with Cobas 4800 (HPV16/18 plus 12 other hrHPV), the infection rates for HPV16, HPV18, and other 12 hrHPV subtypes were 11.11%, 2.22%, and 82.22%, respectively. Histopathological examination confirmed that 137 of the 387 (35.40%) patients with ASC-H had high-grade squamous intraepithelial lesion or worse (HSIL+), with seven HSIL+ lesions testing negative for HPV. Low-grade squamous intraepithelial lesions (LSIL) were identified in 119 cases (30.75%), including 15 that were negative for HPV. There was no significant difference in HSIL+ positivity observed between the two screening groups, whereas the opportunistic screening group showed a significantly higher positivity rate for LSIL compared with the organized cervical screening group (*p*<0.01).

**Conclusion:**

ASC-H indicates a significant risk of HSIL+, particularly in younger women, regardless of interpretation (AI-assisted or manual). These patients require timely evaluation and suitable treatment, as well as careful follow-up to minimize the risk of missed diagnoses of precancerous lesions and early cervical cancer.

## 1 Introduction

Cervical cancer is the most prevalent malignant tumor of the female reproductive tract, ranking fourth in terms of incidence and mortality rates worldwide (1). In 2024, an estimated 144,900 new cases of cervical cancer were recorded in China, corresponding to an incidence rate of 13.96 per 100,000 females and ranking fifth in incidence among all gynecological malignancies. Approximately 49,500 deaths were attributed to cervical cancer, with a mortality rate of 4.0 per 100,000 individuals, making it the sixth leading cause of cancer-related death among women (2). Therefore, cervical cancer poses a substantial public health burden on the physical health of women.

Cervical cancer screening modalities consist of opportunistic screening and organized population-based screening. Currently, cytology testing is among the main detection methods for cervical cancer screening. The 2001 Bethesda System continues to serve as the standard framework for reporting cervical cytology results. Among the subtypes of atypical squamous cells (ASC) (3), atypical squamous cells–cannot exclude high-grade squamous intraepithelial lesion (ASC-H) carries a higher risk of high-grade cervical lesions compared with ASC of undetermined significance (ASC-US) (4, 5). The objective of this retrospective study was to analyze the correlation among ASC-H cytological findings, high-risk human papillomavirus (hrHPV) status, and histopathological outcomes in two cervical screening groups, with the aim of improving management strategies for patients diagnosed with ASC-H through cytology testing.

## 2 Materials and methods

### 2.1 Data source

We retrieved data of patients who underwent a cervical ThinPrep cytology test (LCT) at the pathology department of Ganzhou Maternal and Child Health Hospital (Ganzhou, China) from January 2019 to December 2025. Of the 171,688 cases, 31,100 cases were included in the organized screening group and 140,588 cases were classified into the opportunistic screening group. A total of 663 cases (120 and 543 from the organized and opportunistic screening groups, respectively) tested cytologically positive for ASC-H. Among these patients with ASC-H, 387 subsequently underwent human papillomavirus (HPV) testing and follow-up cervical histopathological examination, with an average age of 41 years (range: 17–75 years, mean ± standard deviation [mean ± SD]: 40 ± 11.54 years). Among them, 73 patients underwent organized screening, with an average age of 50 years (range: 35–64 years, mean ± SD: 50.48 ± 8.20 years). The remaining 314 underwent opportunistic screening, with an average age of 39 years (range: 17–75 years, mean ± SD: 39.34 ± 11.19 years).

The inclusion criteria were as follows: 1) no sexual intercourse and vaginal medication within 48h prior to sampling; 2) no history of cervical or total hysterectomy; 3) absence of severe systemic comorbidities; 4) no pregnancy at screening; 5) no active menstruation during specimen collection; and 6) voluntary acceptance of organized cervical cancer screening.

These clinical data were extracted from standardized clinical intake forms that are routinely completed by the examining gynecologist during the patient encounter. The forms include standardized questions about recent sexual activity, medication use, menstrual status, and pregnancy history as part of routine clinical practice. However, the completeness and accuracy of these records depend on the original documentation quality, which is a limitation inherent to retrospective studies.

### 2.2 Ethical approval

The retrospective study was carried out in accordance with the Declaration of Helsinki. The research protocol was approved by the Ethics Committee of Maternal and Child Health Hospital of Ganzhou City (Approval No.2026(47); Approval Date: July 24, 2026). Since all clinical data were anonymized without private identifiable information, written informed consent was waived by the ethics committee.

### 2.3 Methods

#### 2.3.1 Cervical cell samples

Cervical cell samples were collected by experienced gynecologists. A vaginal speculum was inserted to expose the cervix, secretions around the cervical os were gently wiped away. The sampling brush was inserted into the cervical canal and rotated clockwise five times. Subsequently, the brush head was placed into dedicated cell preservation solution, labeled with patient information, and delivered for cytological testing.

#### 2.3.2 HPV detection

HPV genotyping was performed via two separate detection systems: HPV23 genotyping detection (Taipu Biological Science Co., Ltd., Xiamen, China), including 17 hrHPV subtypes (HPV16, 18, 31, 33, 35, 39, 45, 51–53, 56, 58, 59, 66, 68, 73, 82) and six low-risk HPV subtypes (HPV6, 11, 42, 44, CP8304); and HPV16/18 plus other 12 types of hrHPV detection (Cobas 4800 system; Roche Diagnostics, New Jersey, USA).

#### 2.3.3 LCT testing and diagnosis

Artificial intelligence-assisted (AI-assisted) LCT diagnosis was used for organized screening. AI evaluation was based on the Jiangfeng Bio-AI two-cancer screening information system (version number: 1.3.3.2), using digital image scanning technology; the whole process was operated according to instructions provided by the developer. The AI system first locates abnormal cells on whole-slide images via YOLOX object detection, then extracts global statistical features and performs slide-level TBS classification using a random forest model. The training dataset is sourced from KFBIO multi-center database, which contains over 100,000 cervical liquid-based cytology WSIs collected from more than 30 hospitals nationwide. Stratified quality control is implemented after AI interpretation: 10% of slides with high-confidence NILM results are randomly selected for manual re-review, and all slides classified as positive by AI are mandatorily reviewed by cytopathologists, who can rapidly identify lesions through coordinate markers generated by the AI system. Subsequently, LCT slides obtained through opportunistic screening were processed with automated staining. The diagnostic criteria outlined in The 2001 Bethesda System of the National Cancer Institute were used in this analysis (3). The reported results included: no lesions (negative for intraepithelial lesion or malignancy), ASC-US, ASC-H, low-grade squamous intraepithelial lesion (LSIL), high-grade squamous intraepithelial lesion (HSIL), squamous cell carcinoma, and atypical glandular epithelial cells. Results ≥ASC-US were cytological abnormality.

#### 2.3.4 Histopathological examination

Histopathological specimens were obtained through cervical biopsy, loop electrosurgical excision procedure, or hysterectomy. All tissue samples were fixed in formaldehyde solution and delivered to the pathology department. Two experienced pathologists evaluated all slides. They reviewed the slides with access to the AI results, as the assessment was not blinded. This approach aimed to more accurately reflect real clinical scenarios in which artificial intelligence assisted diagnosis. The categories of pathological diagnosis included normal or inflammation, LSIL, HSIL, adenocarcinoma *in situ*, and cervical cancer.

### 2.4 Statistical analysis

Statistical analyses were performed using SPSS version 24.0 software (IBM Corp., Armonk, NY, USA). Enumeration data were presented as numbers (percentages). Intergroup comparisons were conducted via the χ^2^ test; and a *p*-value <0.05 denoted a statistically significant difference.

Binary multivariable logistic regression was performed to assess the age difference between the two screening groups influenced the comparison of HSIL+ detection rates. The dependent variable was histologically confirmed HSIL+ (HSIL or CC). Independent covariates included screening modality (AI-organized vs manual opportunistic screening), continuous age, and three-category hrHPV status (HPV-negative, other hrHPV, HPV16/18).). All predictors were forced into the model via the Enter method. Regression coefficient (B), standard error (SE), Wald χ², p-value, odds ratio and adjusted odds ratios (OR) with 95% confidence intervals (CIs) were reported. Model fitness was evaluated via −2 Log likelihood, Nagelkerke R², and the Hosmer-Lemeshow goodness-of-fit test; and a *p*-value>0.05 indicated acceptable calibration.

To evaluate potential selection bias caused by incomplete follow-up, baseline demographic and screening characteristics were compared between ASC-H patients with complete HPV and histological follow-up (n=387) and those lost to follow-up (n=276). Continuous age was compared using independent samples t-test; categorical age strata and screening type were compared using χ^2^ test; and a *p*-value>0.05 indicated balanced baseline distribution between the two subgroups.

## 3 Results

### 3.1 ASC-H positivity rate and hrHPV infection rate

**Figure 1:**
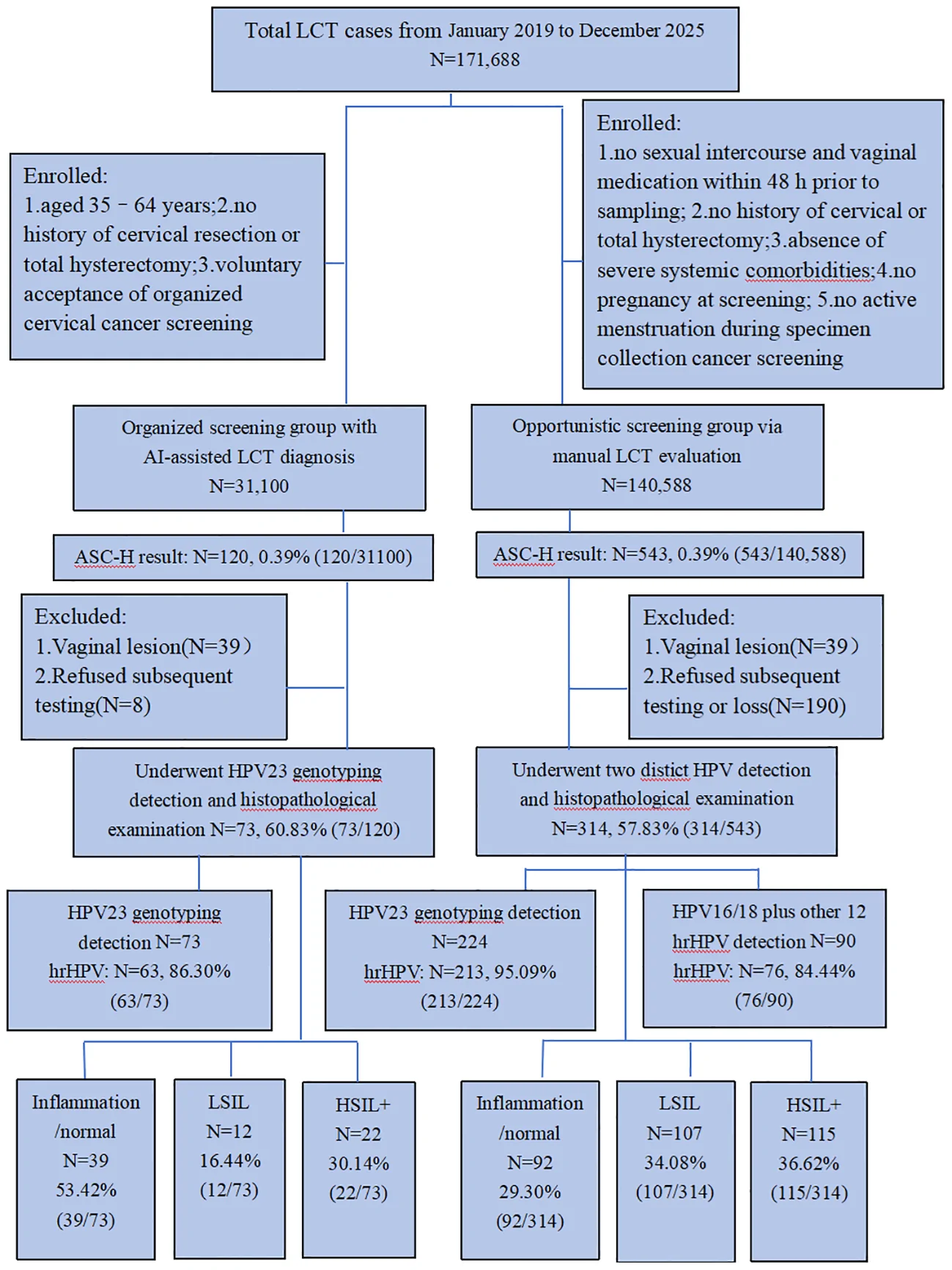
Flowchart of study design. AI, artificial intelligence; ASC-H, atypical squamous cells–cannot exclude high-grade squamous intraepithelial lesion; HPV, human papillomavirus; hrHPV, high-risk human papillomavirus; HSIL, high-grade squamous intraepithelial lesions; HSIL+, HSIL or worse; LSIL, low-grade squamous intraepithelial lesions; LCT, ThinPrep cytology test.

A total of 171,688 cervical LCT cases were included in the analysis, consisting of 31,100 and 140,588 cases from organized and opportunistic screening, respectively. Of those, 663 patients were diagnosed with ASC-H (0.39%), and the positivity rate for ASC-H in the two screening groups was also 0.39%, with 120 cases in the organized screening group (11.28% of all ASC cases) and 543 cases in the opportunistic screening group (9.08% of all ASC cases). Among these 663 patients with ASC-H, 58.37% (387 cases) underwent both HPV genotyping and subsequent cervical histopathological examination. HPV testing was performed via two platforms: 297 samples were tested for 23-type HPV and 90 samples were tested for HPV16/18 plus other 12 hrHPV. The overall HPV infection rate was 84.00% (325 cases) (**Figure 1**).

Among all 663 ASC-H patients, 387 (58.37%) completed paired hrHPV testing and cervical histological biopsy, while the remaining 276 (41.63%) were lost to follow-up. All 387 ASC-H patients with paired histology were divided into two distinct subgroups for separate HPV detection: 297 cases underwent HPV23 genotype detection, while 90 cases underwent HPV16/18 plus other 12 hrHPV detection. The observed differences in hrHPV subtype prevalence between these subgroups resulted from variations in population composition, not from discrepancies in the performance of the two testing methods.

Baseline age and screening type were comparable between followed and lost patients. No significant differences were observed between the two groups in age distribution or screening type (all *p*> 0.05) (**Supplementary Table 1**), suggesting that selection bias due to loss to follow-up may be limited.

Among the 297 patients who underwent HPV23 genotyping, the HPV infection rate was 80.81% (240 cases). When stratified by age, the HPV infection rate in individuals aged <50 years was 79.34%, primarily including single HPV infection cases (51.17%). The HPV infection rate in individuals aged ≥50 years was 85.52%, primarily including multiple HPV infection cases (46.43%). The difference in HPV infection rate between the two age groups was statistically significant (*p*<0.05) (**Table 1**).

**Table 1:** Age-stratified HPV infection rates and distribution of single or multiple hrHPV infections in patients diagnosed with ASC-H through cytology testing (23 types of HPV).

Age	Number of cases	Single hrHPV type n(%)(95%CI)	Multiple hrHPV types n(%)(95%CI)	Total HPV n(%)(95%CI)	χ²	*p*-value
<50 years	213	109 (51.17) (44.50-57.81)	60 (28.17) (22.56-34.56)	169 (79.34) (73.41-84.24)	9.04	0.011
≥50 years	84	32 (38.10) (28.45-48.78)	39 (46.43) (36.15-57.02)	71 (84.52) (75.30-90.73)		
Total	297	141 (47.47) (41.86-53.15)	99 (33.33) (28.22-38.88)	240 (80.81) (75.95-84.88)		

Data represent number (%), unless otherwise indicated. Values in square brackets are 95% Wilson confidence intervals for the proportion.

ASC-H, atypical squamous cells–cannot exclude high-grade squamous intraepithelial lesion; HPV, human papillomavirus; hrHPV, high-risk human papillomavirus; CI, confidence interval.

In HPV23 genotyping cohort(n=297), the HPV positivity rate was 89.04% in the organized screening group (65/73 patients) and 78.13% in the opportunistic screening group (175/224 patients). Among patients aged <50 years, the multiple HPV infection rate was higher in the organized screening group than the opportunistic screening group, whereas the single HPV infection rate was higher in the opportunistic screening group compared with the organized screening group; however, the difference was not statistically significant (*p>*0.05). In those aged ≥50 years, the HPV positivity rate was higher in the organized screening group than the opportunistic screening group, and the multiple infection rate of HPV was higher in the organized screening group compared with the opportunistic screening group, reaching statistical significance (*p*<0.05) (**Table 2**). In HPV16/18 plus other 12 hrHPV subtypes cohort(n=90), the HPV positivity rate was 94.44% (85cases). The positivity rates of the other 12 hrHPV in patients aged <50 years and ≥50 years were 81.43% and 80.00%, respectively, and this difference was not statistically significant. Due to the lack or low number of HPV16/18-positive samples in the two age groups, statistical analysis was not performed (**Table 3**).

**Table 2:** Screening methods and age-stratified positivity rates for HPV and distribution of single or multiple hrHPV infections in patients with ASC-H (23 types of HPV).

			Type of hrHPV				
Age (years)	Screening methods	Number of cases	Single n(%)(95%CI)	Multiple n(%)(95%CI)	Total HPV cases n(%)(95%CI)	χ²	*p*-value
<50						5.77	0.06
	Organized	33	13 (39.39) (24.68-56.32)	15 (45.45) (29.84-62.01)	28 (84.85) (69.08-93.35)		
	Opportunistic	180	96 (53.33) (46.05-60.48)	45 (25.00) (19.24-31.80)	141 (78.33) (71.76-83.73)		
≥50						8.70	0.01
	Organized	40	12 (30.00) (18.07-45.43)	25 (62.50) (47.03-75.78)	37 (92.50) (80.14-97.42)		
	Opportunistic	44	20 (45.45) (31.71-59.93)	14 (31.82) (31.71-59.93)	34 (77.27) (63.01-87.16)		

Data represent number (%), unless otherwise indicated. Values in square brackets are 95% Wilson confidence intervals for the proportion.

ASC-H, atypical squamous cells–cannot exclude high-grade squamous intraepithelial lesion; HPV, human papillomavirus; hrHPV, high-risk human papillomavirus; CI, confidence interval.

**Table 3:** Age-stratified positivity rates for HPV in patients diagnosed with ASC-H through cytology testing (HPV16/18 and other 12 hrHPV detection).

Age (years)	Number of cases	HPV16 n(%)(95%CI)	HPV18 n(%)(95%CI)	Other 12 hrHPV n(%)(95%CI)	Total
<50	70	9 (12.86) (6.91-22.66)	0	57 (81.43) (70.77-88.81)	66 (94.29) (86.21-97.76)
≥50	20	1 (5.00) (0.89-23.61)	2 (10.0) (2.79-30.10)	16 (80.00) (58.40-91.93)	19 (95.00) (76.39-99.11)
Total	90	10 (11.11) (6.15-19.26)	2 (2.22) (0.61-7.74)	73 (81.11) (71.82-87.86)	85 (94.44) (87.65-97.60)

Data represent number (%), unless otherwise indicated. Values in square brackets are 95% Wilson confidence intervals for the proportion.

ASC-H, atypical squamous cells–cannot exclude high-grade squamous intraepithelial lesion; HPV, human papillomavirus; hrHPV, high-risk human papillomavirus.

CI, confidence interval.

### 3.2 hrHPV genotyping for ASC-H cytology

**Figure 2:**
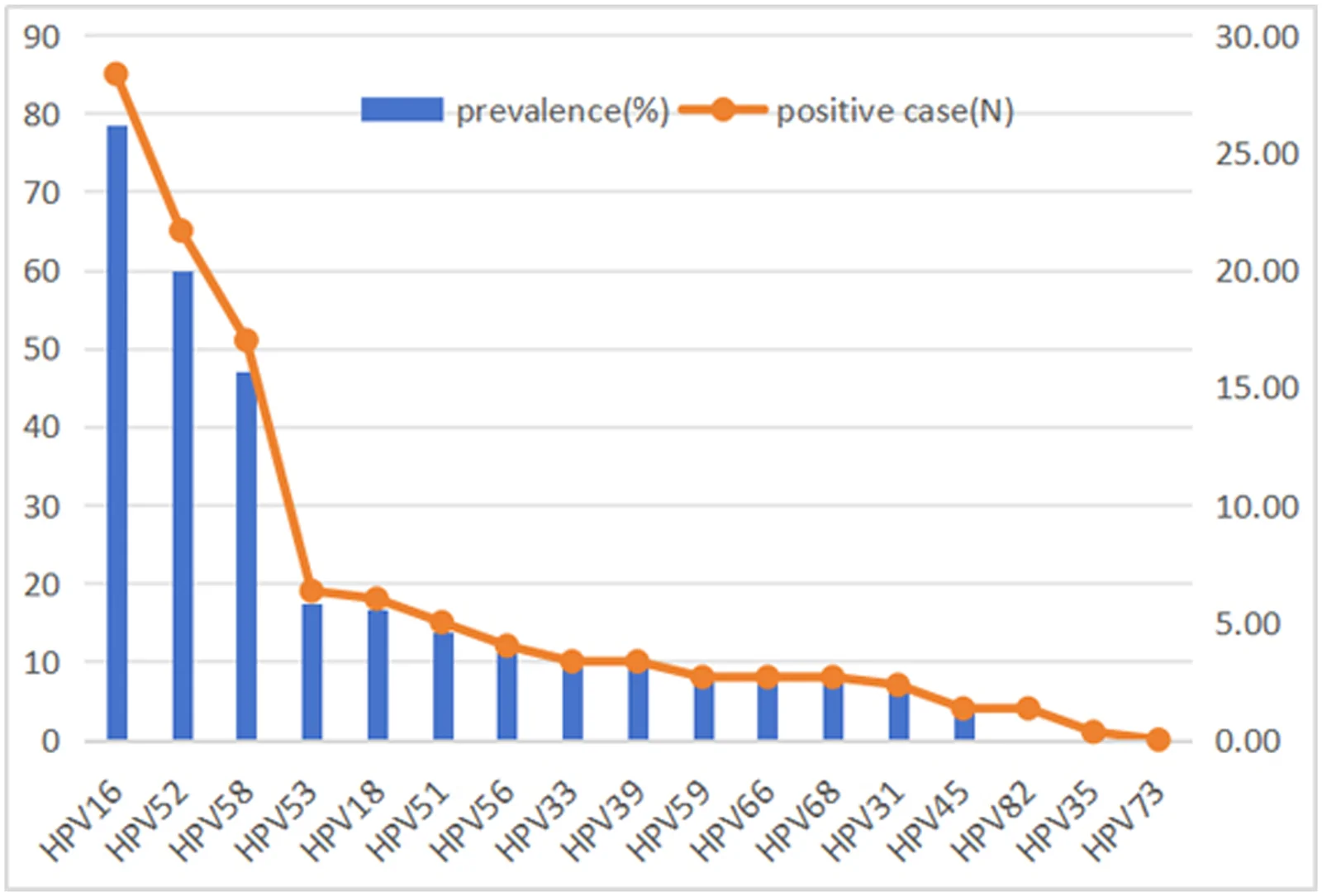
Distribution of any 16 hrHPV genotypes in 297 women diagnosed with ASC-H through cytology testing (HPV23 genotyping detection). ASC-H, atypical squamous cells–cannot exclude high-grade squamous intraepithelial lesion; HPV, human papillomavirus; hrHPV, high-risk human papillomavirus.

Among the 297 ASC-H cases tested with 23-type HPV genotyping, HPV16 (28.62%), HPV52 (21.89%), and HPV58 (17.17%) were the most prevalent hrHPV genotypes. In contrast, HPV45 (1.35%), HPV82 (1.35%), and HPV35 (0.34%) exhibited the lowest positivity rates, while HPV73 was not detected (**Figure 2**).

**Figure 3:**
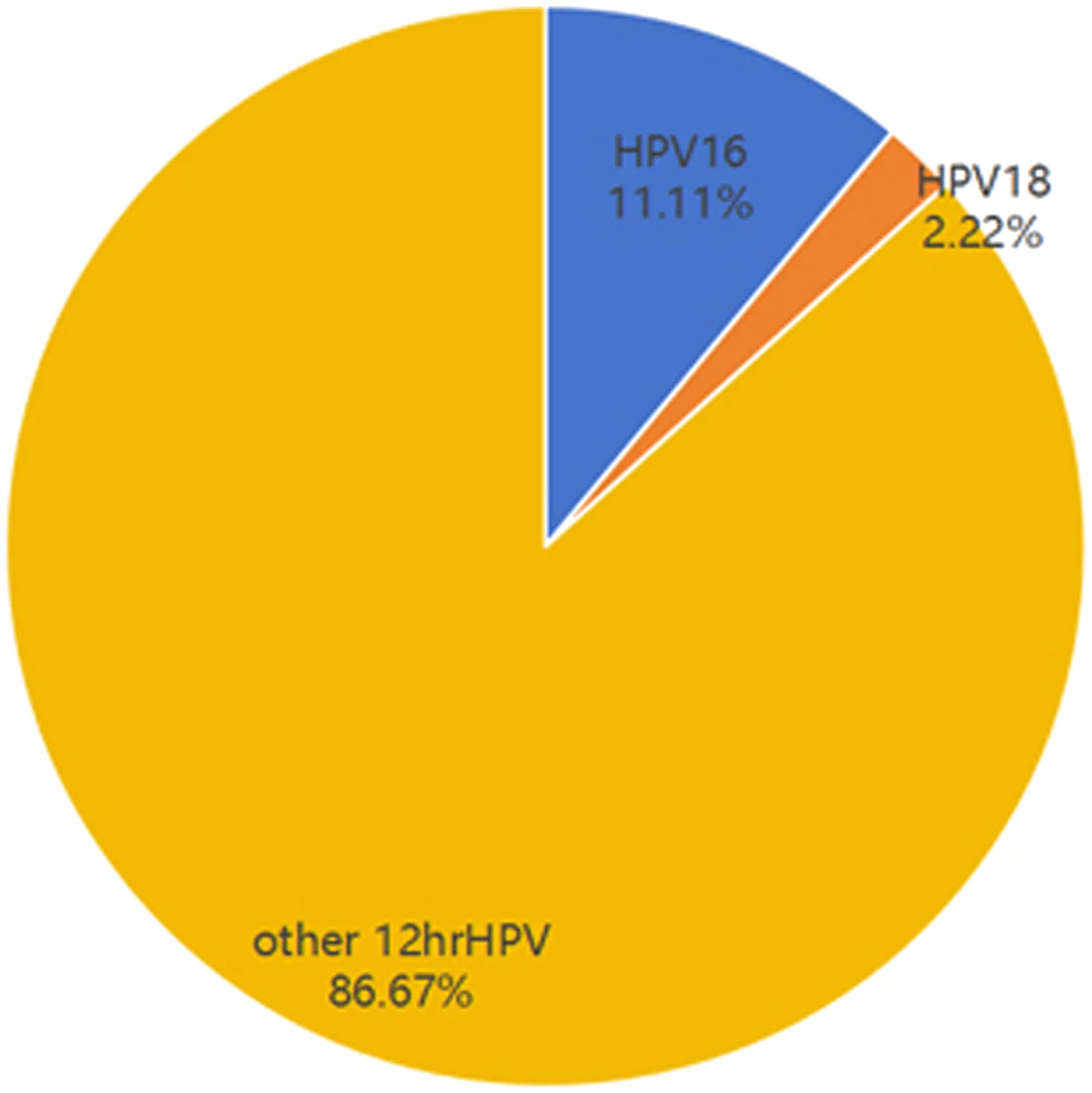
Distribution of 90 women diagnosed with ASC-H through cytology testing (HPV16/18 plus other 12 hrHPV detection). ASC-H, atypical squamous cells–cannot exclude high-grade squamous intraepithelial lesion; HPV, human papillomavirus; hrHPV, high-risk human papillomavirus.

Of the 90 patients diagnosed through HPV16/18 plus other 12 hrHPV subtypes detection, 10 cases were infected with HPV16 (11.11%), two cases were infected with HPV18 (2.22%), and 73 cases were infected with other 12 hrHPV genotypes (81.11%), excluding patients with concurrent HPV16/18 infection (**Figure 3**).

The positive rate of HPV16 varied significantly between the two HPV testing subgroups: 28.62% in the HPV23 genotype detection group and 11.11% in the HPV16/18 plus other 12 hrHPV detection group. This difference arose because the two cohorts consisted of independent, non-overlapping populations with distinct proportions of participants from AI-assisted screenings and opportunistic screenings. Consequently, the disparity did not indicate a difference in the detection capabilities of the two reagents. Therefore, subsequent analyses excluded cross-cohort comparisons of single hrHPV subtype positivity.

### 3.3 ASC-H and histopathological findings

Among 387 patients with ASC-H who underwent histopathological follow-up within 6 months, abnormal lesions (low-grade squamous intraepithelial lesion or worse [LSIL+]) were identified in 256 cases (LSIL: n=119, 30.75%; HSIL+: n=137, 35.40% [HSIL: n=130; cervical cancer: n=7]) (**Table 4**). Among the 387 cases of ASC-H, 66 cases were negative for hrHPV, while 21 cases (31.82%) had histopathological abnormalities (LSIL: n=15; HSIL: n=3; and cervical cancer: n=3).

**Table 4:** Detailed breakdown of the number of cases by age in the two screening groups.

Age (years)	Screening methods	LSIL n(%)(95%CI)	HSIL+ n(%)(95%CI)	Inflammation/normal n(%)(95%CI)	Total
<50		100 (35.34) (30.00-41.07)	105 (37.10) (31.68-42.87)	78 (27.56) (22.68-33.04)	283
	Organized	8 (24.24) (12.83-41.02)	12 (36.36) (22.19-53.38)	13 (39.39) (24.68-56.32)	33
	Opportunistic	92 (36.80) (31.06-42.94)	93 (37.20) (31.44-43.34)	65 (26.00) (20.96-31.77)	250
≥50		19 (18.27) (12.02-26.78)	32 (30.77) (22.72-40.19)	53 (50.96) (41.49-60.36)	104
	Organized	4 (10.00) (3.96-23.05)	10 (25.00) (14.19-40.19)	26 (65.00) (49.51-77.87)	40
	Opportunistic	15 (23.44) (14.75-35.13)	22 (34.38) (23.92-46.60)	27 (42.19) (30.87-54.39)	64
Total		119 (30.75) (26.36-35.52)	137 (35.40) (30.80-40.29)	131 (33.85) (29.31-38.70)	387

Data represent number (%), unless otherwise indicated. Values in square brackets are 95% Wilson confidence intervals for the proportion.

HSIL, high-grade squamous intraepithelial lesion; HSIL+, HSIL or worse; LSIL, low-grade squamous intraepithelial lesion; CI, confidence interval.

Among the 73 patients in the organized screening group, 39 (53.42%), 12 (16.44%), and 22 (30.14%) were normal or had chronic cervicitis, LSIL, and HSIL+, respectively. Among the 314 patients in the opportunistic screening group, 92 (29.30%), 107 (34.08%), and 115 (36.62%) were normal or had chronic cervicitis, LSIL, and HSIL+, respectively. The rates of normal/chronic cervicitis and LSIL differed significantly between the organized screening and opportunistic screening groups (*p*<0.01); nonetheless, the difference in the detection rates of HSIL+ was not statistically significant (*p*=0.34) (**Table 5**).

**Table 5:** Detailed breakdown of the number of cases by category in the two screening groups.

Screening methods	LSIL n(%)(95%CI)	HSIL+ n(%)(95%CI)	Inflammation/normal n(%)(95%CI)	Total
Organized	12 (16.44) (9.66-26.57)	22 (30.14) (20.82-41.44)	39 (53.42) (42.10-64.41)	73
Opportunistic	107 (34.08) (29.05-39.48)	115 (36.62) (31.49-42.08)	92 (29.30) (24.54-34.56)	314
*p****-***value	<0.01	0.34	<0.01	

Data represent number (%), unless otherwise indicated. Values in square brackets are 95% Wilson confidence intervals for the proportion.

HSIL, high-grade squamous intraepithelial lesion; HSIL+, HSIL or worse; LSIL, low-grade squamous intraepithelial lesion; CI, confidence interval.

### 3.4 Histologic findings associated with hrHPV genotype

To assess the risk of lesions associated with distinct hrHPV genotypes, we compared histopathological outcomes between patients with ASC-H who were positive for HPV16/18 and those who were positive for other hrHPV subtypes across the two screening groups (i.e., a total of 314 patients with ASC-H).

In the 107 patients who were positive for HPV16/18, the LSIL detection rate was 28.04% (n=30), with no case of LSIL identified in the organized screening group. The HSIL+ detection rate reached 53.27% (n=57; organized screening group: n=7, opportunistic screening group: n=50). Among the 207 patients who were positive for other hrHPV subtypes, the LSIL detection rate was 34.78% (n=72; organized screening group: n=11, opportunistic screening group: n=61). The HSIL+ detection rate was 35.75% (n=74; organized screening group: n=13, opportunistic screening group: n=61). Patients with HPV16/18 positivity exhibited a significantly higher HSIL+ detection rate than those infected with other hrHPV subtypes (*p*<0.01). A statistically significant intergroup difference was also observed for the LSIL detection rates (*p*<0.05) (**Table 6**).

**Table 6:** Detailed breakdown of histological examination results and case numbers by HPV category in the two screening groups.

HPV subtype	Screening methods	LSIL n(%)(95%CI)	HSIL+ n(%)(95%CI)	Inflammation/normal n(%)(95%CI)	Total
HPV16/18		30 (28.04)(20.40-37.20)	57 (53.27) (43.87-62.45)	20 (18.69) (12.44-27.11)	107
	Organized	0 (0)	7 (46.67) (24.81-69.88)	8 (53.33) (30.12-75.19)	15
	Opportunistic	30 (32.61) (23.89-42.72)	50 (54.35) (44.20-64.15)	12 (13.04) (7.62-21.43)	92
Other hrHPV		72 (34.78) (28.63-41.49)	74 (35.75) (29.53-42.48)	61 (29.47) (23.68-36.01)	207
	Organized	11 (22.92) (13.31-36.54)	13 (27.08) (16.57-41.00)	24 (50.00) (36.39-63.61)	48
	Opportunistic	61 (38.36) (31.17-46.11)	61 (38.36) (31.17-46.11)	37 (23.27) (17.38-30.42)	159
Total		102 (32.48) (27.54-37.85)	131 (41.72) (36.40-47.24)	81 (25.80) (21.27-30.91)	314

Data represent number (%), unless otherwise indicated. Values in square brackets are 95% Wilson confidence intervals for the proportion.

HPV, human papillomavirus; HSIL, high-grade squamous intraepithelial lesion; HSIL+, HSIL or worse; LSIL, low-grade squamous intraepithelial lesion; CI, confidence interval.

### 3.5 Age-adjusted multivariable logistic regression analysis

To further verify whether age imbalance confounded the comparison between AI-assisted organized screening and manual opportunistic screening, a secondary regression model incorporating continuous age was conducted. We found that (1):After adjusting for age and HPV status, the HSIL+ detection rate remained comparable between the AI-assisted and manual interpretation groups, confirming the robustness of the main conclusion (2).Age was not a significant predictor of HSIL+ (3).Patients with other high-risk HPV types had 5.5 times the odds of HSIL+ compared to HPV-negative patients, and patients with HPV16/18 had 11.5 times the odds of HSIL+ compared to HPV-negative patients. The above results indicated that age difference does not affect the primary finding. The main driver of HSIL+ is HPV infection status, not the screening modality. The unadjusted OR for screening method was identical (OR=0.75, 95% CI: 0.43-1.29, *p*=0.298), confirming that age adjustment did not substantially alter the result. The Hosmer-Lemeshow test demonstrated good model calibration (χ^2^ =8.73, df=8, *p*=0.366) (**Supplementary Table 2**).

## 4 Discussion

Cervical cancer is a common malignant tumor posing a serious threat to the health of women. Standardized cervical cancer screening can maximize the frequency of examination for eligible women, thereby facilitating the early identification of precancerous cervical lesions and early invasive cervical carcinoma (6). According to data from 2023–2024, the coverage rate of cervical cancer screening for eligible women in China has reached 51.5%, and the incidence of cervical cancer is increasing (7). In some regions with established population-based organized cervical cancer screening programs, the incidence of cervical cancer has decreased markedly by approximately 70% (8). Typically, organized screening is more effective and efficient in reducing the burden of cervical cancer than opportunistic screening (9). Organized screening features broad coverage and a lower risk of missed diagnosis, while opportunistic screening boasts targeted recruitment and favorable patient compliance (10).

Due to the large population and uneven distribution of urban and rural population in China, the combination of organized screening and opportunistic screening is an effective means to improve the efficiency of cervical cancer screening. The current screening programs include HPV primary screening, cytology primary screening, and HPV and cytology combined screening. The cytological characteristics of ASC-H are suggestive of HSIL; nevertheless, they are insufficient for reaching a definitive diagnosis. The cytological report of ASC-H is extremely rare and the diagnosis is subjective. Thus, there is a marked difference in diagnosis between different doctors (reporting rate: 0.22–1.09%). ASC-H accounts for approximately 5–10% of all diagnosed ASC cases (11, 12). In our study, the ASC-H reporting rate was 0.39% in both organized screening or opportunistic screening populations, while the proportion of ASC-H among all ASC cases was 11.28 and 9.06%, respectively. This proportion was slightly higher in the organized screening group than the opportunistic screening group. This difference may be attributed to the more relaxed in the interpretation of suspicious high grade lesion cells using AI-assisted LCT diagnosis.

The overall hrHPV infection rate among patients with ASC-H is approximately 80% (12, 13). In our study, the total HPV infection rate in patients with ASC-H reached 84.00%. HPV16, HPV52, and HPV58 were the predominant hrHPV subtypes detected in ASC-H. The data were similar to the results regarding the main hrHPV infection subtypes in the domestic research population; however, they differed slightly from the global and European/American pattern, where HPV16, HPV18, and HPV31 exhibit the highest rates (14–16). These findings indicate notable regional disparities in hrHPV infection profiles. When comparing histopathological LSIL+ results between patients with ASC-H who were positive for HPV16/18 and those infected with other hrHPV subtypes, we observed a significantly higher HSIL+ detection rate in the former group relative to the latter group. Therefore, clinicians should prioritize monitoring for HPV16, HPV18, HPV52, and HPV58 genotypes.

Substantial divergence in HPV16 positivity emerged between patients who underwent HPV23 genotype detection and those who underwent HPV16/18 plus other 12 hrHPV detection. This discrepancy resulted entirely from the non-overlapping design of the HPV testing subgroups in our retrospective cohort. Specifically, the two sets of samples originated from distinct screening branches, which differed in both age distribution and screening methods, rather than from parallel testing of the same individuals. Consequently, the subtype prevalence data from the two platforms could not be directly compared. For this reason, we analyzed hrHPV related pathological risks within each cohort separately, without conducting cross-assay comparisons.

In this study, LSIL and above lesions were confirmed by histopathology in 5.68% of patients with HPV-negative ASC-H, implying that the clinical management of patients with ASC-H should not depend solely on hrHPV status. Adjunctive testing modalities including p16/Ki-67 dual staining, cervical cell methylation assays, and colposcopy should also be applied. Diagnostic cervical biopsy is recommended when indicated to reduce missed cervical lesions and prevent overtreatment.

Previous studies have demonstrated that high-grade cervical lesions tend to occur more frequently among younger women, and positive histopathological findings are strongly associated with younger age (11). In our population, HSIL and above lesions accounted for 35.40% of histologically followed ASC-H cases. The HSIL+ detection rate was markedly higher in patients aged <50 years compared with those aged ≥50 years (37.10% vs. 30.77%, respectively). No significant intergroup difference in HSIL+ detection rate was observed between the organized screening group (ASC-H diagnosed via AI-assisted LCT) and the opportunistic screening group (ASC-H diagnosed by manual evaluation) (30.14% vs. 36.62%, respectively). The robustness of comparable HSIL+ risk between AI-assisted and manual screening cohorts was validated via two separate multivariable logistic regression models. Both models yielded consistent non-significant associations between screening modality and HSIL+ after full adjustment for age and hrHPV infection. Continuous age did not independently predict HSIL+, demonstrating that the baseline age disparity between the two screening groups did not confound our core comparison conclusion. In contrast, rates of normal/chronic cervicitis and LSIL differed significantly between the two groups. These results indicate that AI-augmented LCT shows similar performance to manual LCT in identifying HSIL+ in this single-center ASC-H cohort after confounding adjustment. Liu et al. demonstrated that the AI screening system outperformed manual screening in accuracy for HSIL, LSIL, and inflammation (82.54% vs. 75.90%, 87.47% vs. 79.41%, respectively), with sensitivity for HSIL also exceeding that of manual screening (67.53% vs. 40.91%) (17). Our findings indicated comparable detection rates of HSIL+ between AI-assisted and manual reading of LCT in this single-center retrospective ASC-H cohort, after controlling for age and hrHPV confounding variables. However, the AI system showed notable limitations in interpreting atrophic, reactive, and crowded glandular cells, resulting in variable detection of LSIL and benign lesions between the two screening methods. Our study found that AI-assisted LCT shows limitations in distinguishing deep-stained crowded cell clusters due to senile atrophy, reactive repair, clustered glandular cells, or true HSIL lesions. Larger datasets for iterative self-learning optimization are required to further enhance the specificity and overall diagnostic quality of the AI system. Although all slides flagged positive by the AI screening system undergo manual physician review, AI-positive outputs may introduce bias in the subjective assessment of the clinician to some degree. This can lead to colposcopy referrals for a subset of false-positive cases. Histopathological HSIL+ was confirmed in 77.1% of LCT HSIL+ cases in our internal data, suggesting favorable diagnostic performance of AI-assisted screening. Further data collection and multicenter investigations were warranted to enhance the capability of AI-assisted LCT diagnosis.

There are some limitations in this study. Firstly, some data are lacking because a proportion of patients with ASC-H chose to undergo follow-up in other hospitals or were lost to follow-up. Although we performed baseline comparison between followed and lost ASC-H patients to quantify selection bias from incomplete follow-up. Age and screening mode were well balanced between the two subgroups, indicating that demographic and screening source imbalance did not substantially distort histological outcome estimates. Nevertheless, this comparison was limited by unavailable hrHPV and histological data for lost patients; we could not verify whether asymptomatic HPV-negative patients were disproportionately lost, so mild residual non-random missing bias (MNAR) cannot be fully excluded. Secondly, we compared baseline characteristics between patients who completed follow-up and those lost to follow-up, and analyzed the selection bias due to loss to follow-up may be limited. However, HPV status and histological outcomes were unavailable for the lost group, which is an inherent limitation of this retrospective study. Thirdly, this was a single-center retrospective study. Future investigations should include larger sample sizes, and multi-center studies should be conducted to increase the generalizability of the findings.

## 5 Conclusion

In summary, the high risk of HSIL+ in subsequent histopathology examination of patients with ASC-H was not significantly different between modalities of cytological diagnosis (i.e., AI-assisted organized screening or opportunistic screening with manual interpretation). Thus, a flexible combination of cervical cancer screening modalities is recommended. Incorporating advanced technologies, such as AI into screening workflows, may facilitates earlier identification of cervical precancerous lesion and invasive cervical cancer, though these findings warrant further validation in prospective multicenter studies.

## Data Availability

The raw data supporting the conclusions of this article will be made available by the authors, without undue reservation.

## Supplementary material

Supplementary Table 1 Comparison of Baseline Characteristics Between Patients with and without Complete Follow-up. SD, standard deviation.

Supplementary Table 2 Multivariable Logistic Regression Analysis of Factors Associated with HSIL+ in Patients with ASC-H. ASC-H, atypical squamous cells–cannot exclude high-grade squamous intraepithelial lesion; HSIL, high-grade squamous intraepithelial lesion; HSIL+, HSIL or worse; HPV, human papillomavirus; B, regression coefficient; SE, standard error; OR, odds ratio; CI, confidence interval.
