# Supplementary material for: Correlation between atypical squamous cells–cannot exclude high-grade squamous intraepithelial lesion and cervical lesions: a retrospective analysis of opportunistic and organized cervical screening populations
Source: Front Oncol. 2026 Aug 14;16:1926183. doi: 10.3389/fonc.2026.1926183 (PMC13521855; doi:10.3389/fonc.2026.1926183)
Supplement: Supplementary file 1 [file Table1.pdf]

Supplementary Table S1. Comparison of Baseline Characteristics Between Patients with and without Complete Follow-up

Characteristic	Followed (n=387)	Lost to follow-up (n=276)	p-value
Age, mean ± SD, years	40.1 ± 12.3	41.5 ± 13.1	0.18
Age group, n (%)			0.24
<50 years	283 (73.1)	192 (69.6)	
≥50 years	104 (26.9)	84 (30.4)	
Screening type, n (%)			0.51
Organized	73 (18.9)	47 (17.0)	
Opportunistic	314 (81.1)	229 (83.0)	

Abbreviation:SD,standard deviation.
