# Supplementary material for: Correlation between atypical squamous cells–cannot exclude high-grade squamous intraepithelial lesion and cervical lesions: a retrospective analysis of opportunistic and organized cervical screening populations
Source: Front Oncol. 2026 Aug 14;16:1926183. doi: 10.3389/fonc.2026.1926183 (PMC13521855; doi:10.3389/fonc.2026.1926183)
Supplement: Supplementary file 2 [file Table2.pdf]

Supplementary Table S2. Multivariable Logistic Regression Analysis of Factors Associated with HSIL+ in Patients with ASC-H

Variable	B	SE	Wald χ^2	p-value	OR	95% CI for OR
Screening method						
Organized vs Opportunistic	-0.283	0.313	0.82	0.366	0.75	0.41-1.39
Age (per year increase)	-0.004	0.010	0.16	0.692	0.996	0.976-1.016
HPV infection category (ref: HPV-negative)						
Other hrHPV (vs negative)	1.704	0.454	14.12	0.0002	5.50	2.26-13.37
HPV16/18 (vs negative)	2.438	0.471	26.80	<0.0001	11.45	4.55-28.81
Constant	-1.501	0.612	6.02	0.014	0.223	-
Model fit						
-2 Log likelihood	458.36					
Nagelkerke R^2	0.124					
Hosmer-Lemeshow χ^2	8.73 (df=8)			0.366		

Abbreviations: ASC- H, atypical squamous cells–cannot exclude high- grade squamous intraepithelial lesionl;HSIL, high-grade squamous intraepithelial lesion; HSIL+, HSIL or worse; HPV, human papillomavirus; B, regression coefficient; SE,standard error; OR,odds ratio; CI, confidence interval.
